# LingZhi oligopeptides amino acid sequence analysis and anticancer potency evaluation†

**DOI:** 10.1039/c9ra10400c

**Published:** 2020-02-27

**Authors:** Jie Liu, Huailing Wang, Qiang Luo, Shuqi Qiu, Zhendan He, Zhigang Liu, Liteng Yang, Xiaoyu Liu, Xizhuo Sun

**Affiliations:** The Third Affiliated Hospital of Shenzhen University, Shenzhen University Shenzhen 518020 China bwordemail@163.com lszuemail@163.com +86-0755-86671914 +86-0755-86671911; School of Medicine, Shenzhen University Shenzhen 518060 China 2958317364@qq.com +86-0755-86671905; Longgang ENT Hospital & Shenzhen ENT Institute Shenzhen 518172 China; Department of Pharmacy, Shenzhen University Shenzhen 518060 China

## Abstract

LingZhi (*Ganoderma lucidum*) has been used as a therapeutic agent for decades, but the antitumor potency of LingZhi oligopeptides (LZOs) was not well explored. In current study, ten novel LZO amino acid sequences were identified, and anticancer potency was evaluated. We found that LZO-3 [EGHGF] significantly triggered A549 cell apoptosis *via* mitochondrial dysregulation, as evidenced by caspases activation, mitochondrial membrane potential collapse, Bcl-2/Bax ratio alteration, and cytochrome c release. Further, the down-regulation of Trx/TrxR reductase and the improvement of the MDM2/p53 state also contributed to the LZO-3-induced cell apoptosis. Notably, our findings provide evidence for the novel potency of LZOs, which could be developed as promising chemotherapeutic agents against lung cancer.

## Abstract 2

The LingZhi (*Ganoderma lucidum*) has been used as a therapeutic agent for decades, but the antitumor potency of LingZhi oligopeptides (LZOs) was not well explored. In current study, ten novel LZOs were amino acid sequence identified and anticancer potency evaluated.

## Graphical abstract

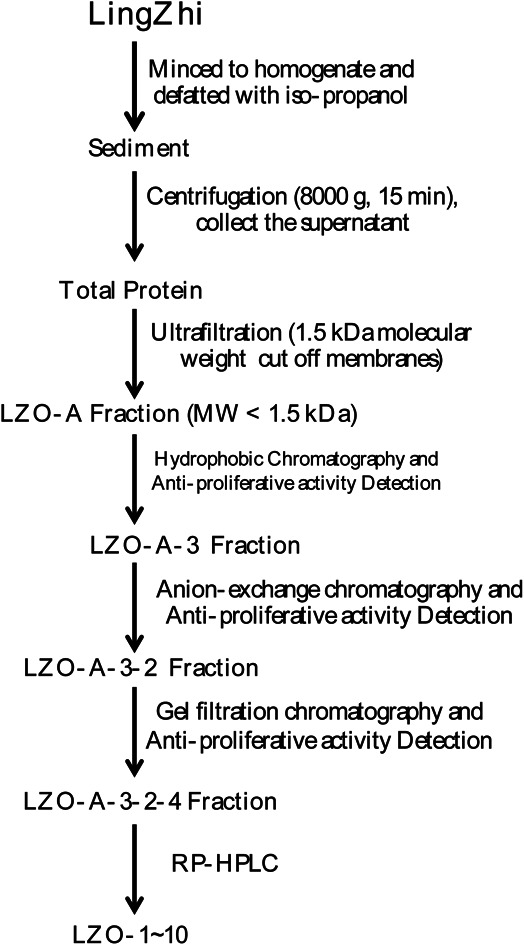

## 1 Introduction

Nowadays, lung cancer is one of the most serious malignant tumors worldwide, with over 1.5 million deaths each year.^1^ Advanced treatments of lung cancer have been achieved over the past two decades, and the use of small-molecule tyrosine kinase inhibitors and immunotherapy have led to unprecedented survival benefits in selected patients.^2,3^ However, the crucial factor that contributes to the current therapy failure includes serious side effects and the emergence of drug resistance to conventional chemotherapy drugs.^4^ Therefore, the development of new agents is urgently needed to improve the outcomes of lung cancer.

In recent years, there have been growing interests in exploring the potential of natural products against tumors. Further, plant-derived natural products have shown promising potency in clinical models.^5^ LingZhi (*Ganoderma lucidum*) is widely used for its anti-inflammatory, anti-LZOterial, and anti-oxidative properties due to its abundant bioactive compounds.^6,7^ In addition, the water extracts and polysaccharides from LingZhi show excellent anti-tumor potency in several tumor-bearing animals.^8^

Natural peptides have gradually become the main substitutes for anti-cancer agents because of their remarkable efficacy, better stability, and lower toxicity.^9^ In the current study, we demonstrate for the first time that LingZhi oligopeptides (LZOs) are effective against human lung cancer cells, and the investigation of the molecular mechanism revealed that LingZhi oligopeptide-3 (LZO-3)-induced apoptosis in A549 cells through ROS accumulation and Δ*Ψ*_m_ collapse *via* caspases activation. Further, the results revealed that LZO-3 inhibited the Trx/TrxR expressions and improved the MDM2/p53 state, which in turn induced the ROS accumulation and apoptosis of A549 cells.

## 2 Materials and methods

### 2.1 Reagents and antibodies

LingZhi (*Ganoderma lucidum*) was acquired from the Ertiantang Pharmacy (Guangzhou, China) and identified by Professor Zhou (Jinan University, Guangzhou, China). The reagents PI and JC-1, RIPA buffer, and RNase were purchased from Beyotime (Shanghai, China). The antibodies were purchased from Cell Signaling Technology (CST, Beverly, MA, USA). Fetal bovine serum (FBS) was purchased from Invitrogen (Thermo Fisher Scientific, USA). All other chemicals were purchased from Sigma or Adamas and were used without any further purification. The tumor and normal cells were obtained from the Cell Bank of the Chinese Academy of Sciences (Shanghai, China).

### 2.2 Isolation and identification of the LZOs

All extraction and separation procedures were performed at 4 °C. The LingZhi (1.25 kg) was minced and stirred for 4 h in the 2500 mL H_2_O/iso-propanol (1 : 15 w/v). Then, the sediment was collected, freeze-dried, and stored at −20 °C. The precipitate (110 g) was dissolved (5%, w/v) in a 0.20 M phosphate buffer solution (PBS, pH 7.3). After centrifugation (8000×*g*, 15 min), the supernatant was collected, freeze-dried, and stored as total protein at −20 °C.

#### 2.2.1 Fractionation by ultrafiltration

The resulting supernatant was fractionated using ultrafiltration with 1 kDa molecular weight (MW) cut-off membranes (Millipore, Hangzhou, China) for the lab scale. Two peptide fractions, called LZO-A (MW < 1 kDa) and LZO-B (MW > 1 kDa), were collected and freeze-dried.

Hydrophobic chromatography: the LZO-A was dissolved in 1.20 M (NH_4_)_2_SO_4_ prepared with 30 mM PBS (pH 7.2) and loaded onto a Phenyl Sepharose CL-4B hydrophobic chromatography column (3.0 cm × 90 cm), which had previously been equilibrated with the above buffer. A stepwise elution was carried out with decreasing concentrations of (NH_4_)_2_SO_4_ (1.20, 0.60, and 0 M) dissolved in 30 mM phosphate buffer (pH 7.2) at a flow rate of 2.0 mL min^−1^. Each fraction was collected at a volume of 50 mL and was monitored at 280 nm. Ten fractions were collected and freeze-dried, and then the anti-proliferative activity against the lung cancer cell lines was detected. The fraction having the strongest anti-proliferative activity was collected and used for anion-exchange chromatography.

#### 2.2.2 Anion-exchange chromatography of LZO-A-3

The LZO-A-3 solution (3 mL, 674.0 mg mL^−1^) was loaded into a DEAE-52 cellulose (Yuanju, Shanghai, China) anion-exchange column (2.2 × 100 cm) pre-equilibrated with deionized water and was stepwise eluted with 2400 mL distilled water, followed by 0.10, 0.60, and 1.20 M (NH_4_)_2_SO_4_ solutions at a flow rate of 2.0 mL min^−1^. Each eluted fraction (60 mL) was collected and detected at 280 nm. Six fractions (A-LZO-3-1 to A-LZO-3-6) were collected and freeze-dried, and then the anti-proliferative activity against the lung cancer lines was detected. The fraction having the strongest anti-proliferative activity was collected and used for gel filtration chromatography.

#### 2.2.3 Gel filtration chromatography of LZO-A-3-2

The LZO-A-3-2 solution (2 mL, 226 mg mL^−1^) was fractionated on a Sephadex G-25 (Sigma-Aldrich, Shanghai, China) column (2.5 × 120 cm) at a flow rate of 1.0 mL min^−1^. Each eluent (80 mL) was collected and monitored at 280 nm, and four fractions (LZO-A-3-2-1 to LZOA-3-2-4) were collected and freeze-dried, and then the anti-proliferative activity against the lung cancer cell lines was detected. The fraction having the strongest anti-proliferative activity was collected and used for reversed phase-high performance liquid chromatography (RP-HPLC).

#### 2.2.4 LZO isolation from LZOA-3-2-4 by RP-HPLC

**Fig. 1:**
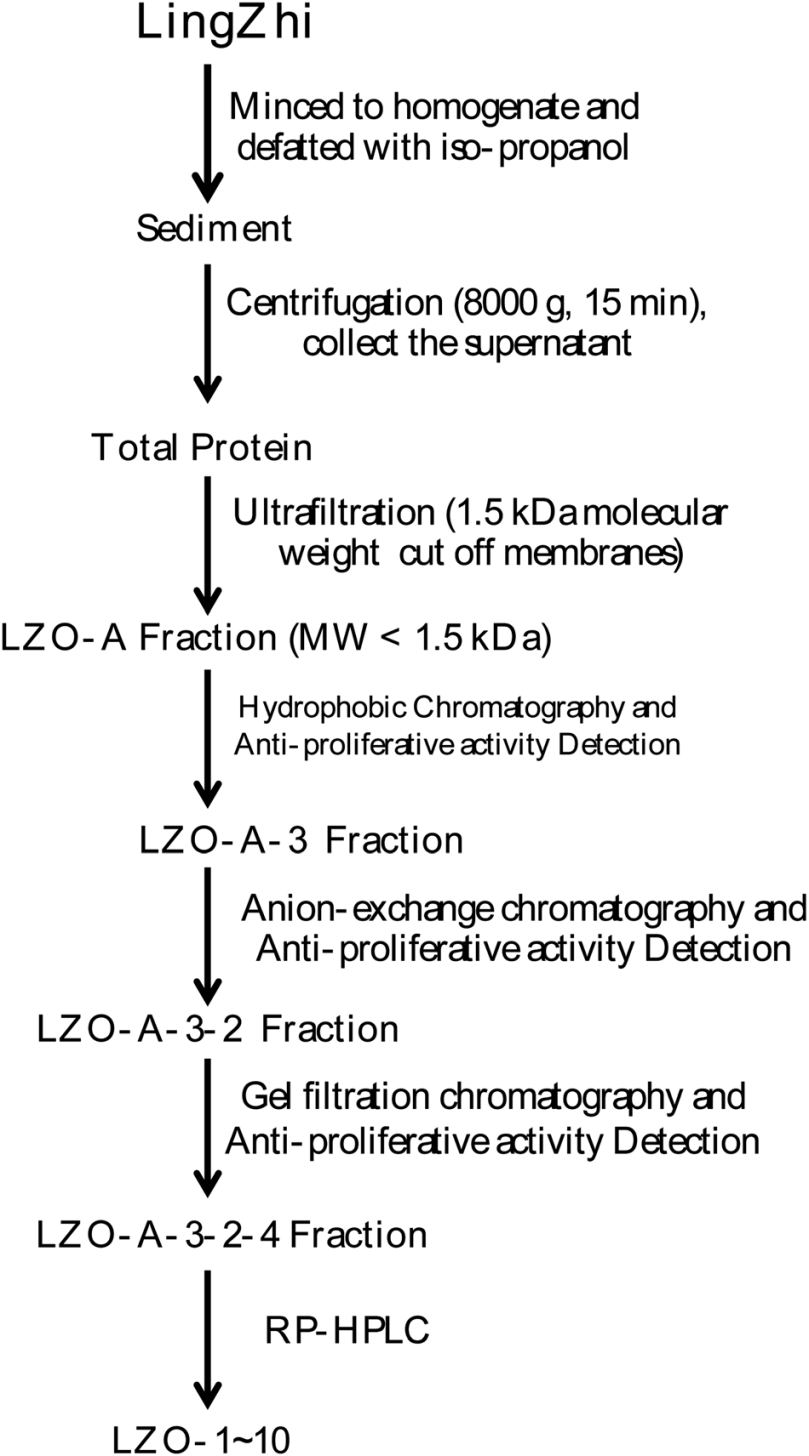
Extraction scheme of LZOs.

LZOA-3-2-4 was finally separated *via* RP-HPLC (Agilent 1200 HPLC) on a Zorbax, SB C-18 column (4.6 × 250 mm, 5 μm). The elution solvent system was composed of water–trifluoroacetic acid (solvent A; 100 : 0.1, v/v) and acetonitrile–trifluoroacetic acid (solvent B; 100 : 0.1, v/v). The peptide was separated using gradient elution from 30% to 80% of solvent B for 60 min at a flow rate of 1.0 mL min^−1^. The detection wavelength and column temperature were set at 280 nm and 20 °C. The eluent was assessed at 280 nm, and after the LZOA-3-2-4 was subjected to HPLC, the LZO-1 (tR 3.1 min), LZO-2 (tR 5.9 min), LZO-3 (tR 7.55 min), LZO-4 (tR 9.73 min), LZO-5 (tR 11.3 min), LZO-6 (tR 19.7 min), LZO-7 (tR 28.7 min), LZO-8 (tR 33.9 min), LZO-9 (tR 46.0 min), and LZO-10 (tR 52.8 min) were afforded. The purity of all the LZOs was detected by HPLC (>97.0%), and the LZOs were dissolved in PBS (10.0 mM). The chromatographs of all the LZOs are summarized in Fig. 1.

### 2.3 Cell culture and MTT assay

Both cancer and normal cells were acquired from the Cell Bank of the Chinese Academy of Sciences (Shanghai, China). A549, NCI-H460, NCI-H23 cancer cells were cultured in a specific medium supplemented with 10% FBS and antibiotics (100 U mL^−1^ streptomycin and 100 U mL^−1^ penicillin) at 37 °C in 5% CO_2_.

The cell viability was assessed with the MTT assay, as previously described.^10^ Briefly, A549 cells (5 × 10^4^) were seeded in a 96-well plate for the initial attachment and incubated with different concentrations of LZOs at various time intervals. After incubation, the medium was removed and the cells were incubated with freshly prepared MTT (1 mg mL^−1^) for 3 h. The formazan salt was dissolved with 100 μL of DMSO, and the absorbance was taken at 570 nm. The same protocol was followed to assess the LZO cytotoxicity in NCI-H460 and NCI-H23 cells. The cytotoxicity of LZOs on human normal pulmonary epithelial cells was assessed by the same method.

### 2.4 Cell cycle and apoptosis analysis

Cell cycle distribution was assessed *via* flow cytometry analysis.^11^ Briefly, after the LZO-3 treatments, the A549 cells were harvested and washed with ice-cold PBS twice. The cell pellet was suspended overnight in 70% ethanol (v/v) at −20 °C. Further, the fixed cells were washed with PBS and stained with PI (50 μg mL^−1^) and RNaseA (100 μg mL^−1^) for 30 minutes without light. The stained cells were then analyzed *via* flow cytometry (FACSCalibur, Franklin Lakes, USA).

The apoptosis of A549 cells was detected through Annexin V/FITC and PI staining according to the manufacturer protocol. Briefly, the A549 were placed in a 6-well plate at a density of 2 × 10^5^ cells per well and treated with LZO-3 for 48 h. Then, the A549 cells were harvested, collected, and apoptosis was detected by Annexin V and PI staining *via* flow cytometry (FACSCalibur, Franklin Lakes, USA).

### 2.5 Measurements of mitochondrial membrane potential (Δ*Ψ*_m_)

The changes in Δ*Ψ*_m_ upon LZO-3 treatments were detected by JC-1 staining according to the manufacturer's protocol (Beyotime, China). Briefly, A549 cells were treated with 0.16 mM of LZO-3 for different time points (0, 6, 12, 24, 48, and 60 h) and different concentrations (0, 0.16, and 0.80 mM) for 48 h. After the treatments, the cells were harvested and washed three times with cold PBS and incubated with 1 mg mL^−1^ of JC-1 at 37 °C for 30 min without light. The supernatant was removed and washed three times with cold PBS, and then assayed *via* the flow cytometric analysis (FACSCalibur, Franklin Lakes, USA). Each treatment was performed in triplicate.

The generation of ROS upon LZO-3 treatments was analyzed *via* the DCF-DA method, as previously described. Briefly, the A549 cells were treated as described above. After the treatments, the A549 cells were harvested, and the ROS level was measured by the ROS assay kit (Beyotime, China) according to the manufacturer's protocol.

### 2.6 Western blotting

A549 cells (3 × 10^6^/dish) were seeded in 10.0 cm culture dishes and cultured overnight. Then, the cells were treated with LZO-3 (0, 0.16, and 0.80 mM) for 48 h. Furthermore, the cells were harvested, and the total protein was collected and measured with the Bradford protein assay to calculate the quantity of protein. Equal amounts of protein were separated on sodium dodecyl sulfate polyacrylamide gel electrophoresis (SDS-PAGE), and were electroblotted to a polyvinylidene difluoride (PVDF) membrane. The immunoblots were blocked with 5% nonfat milk and subsequently incubated with the primary antibody (1 : 1000) at 4 °C overnight, followed by incubation with the peroxidase-conjugated second antibody (1 : 5000) at room temperature for 2 h.^12^ The protein bands were measured, and the β-actin was used as an internal standard of the control process. The blot bands densitometry was analyzed with the ImageJ software.

### 2.7 Statistical analysis

The experiments were repeated at least three times, and results are expressed as mean ± SD. The data were analyzed by the Student *t*-test, and an analysis of the variance (ANOVA) test, followed by a Tukey post-test to determine the significant differences between groups. *p* < 0.05 was considered to be significant.

## 3 Results and discussion

### 3.1 LZO-3 significantly inhibits the growth of lung cancer cells

**Fig. 2:**
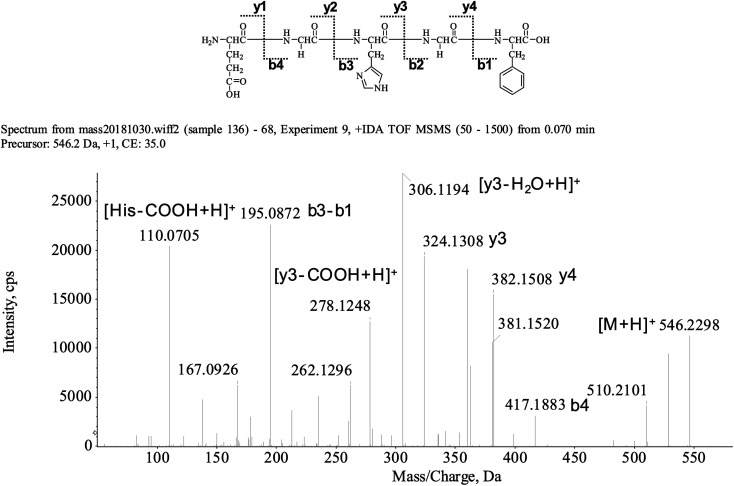
MS spectrum and structure of LZO-3.

The peptide is usually protonated under ESI-MS/MS conditions, and the fragmentations mostly occur at the amide bonds because it was difficult to break the chemical bonds of the side chains at such a low energy. Therefore, the b and y ions were the main fragment ions when the collision energy was <200 eV.^13^ We isolated the LingZhi oligopeptides, and the amino acid sequence was identified *via* the HPLC–ESI-MS analysis. LingZhi oligopeptide-3 (LZO-3) was analyzed by HPLC–ESI-MS for molecular mass determination and peptide characterization (Fig. 2). The ion fragment *m*/*z* 546.2298 was regarded as [M + H]^+^. The ion fragment *m*/*z* 417.1883 was regarded as the b4 ion, while *m*/*z* 382.1508 was regarded as the y4 ion. Subsequently, *m*/*z* 324.1308 was regarded as the y3 ion, and *m*/*z* 306.1194 was regarded as the [y3-H_2_O + H]^+^ ion. The ion with *m*/*z* 278.1248 was the [y3-COOH + H]^+^ ion, *m*/*z* 195.0872 was regarded as the b3-b1 ion, and *m*/*z* 110.0705 was the typical fragment [His-COOH + H]^+^. On this basis, we concluded that the sequence of the peptide was EGHGF. The rest of the LZOs were identified, and the amino acid sequences are listed in Table 1.

**Table 1:** Amino acid sequences and anti-proliferative potency of LZOs in lung cancer cells^a^

LZOs	Amino acid sequences	IC_50_ (mM)		
		A549	NCI-H460	NCI-H23
LZO-1	LYEHGSW	0.42 ± 0.04	0.78 ± 0.06	0.65 ± 0.07
LZO-2	FEHSG	0.29 ± 0.03	0.33 ± 0.03	0.45 ± 0.03
LZO-3	EGHGF	0.16 ± 0.02	0.28 ± 0.03	0.22 ± 0.02
LZO-4	FSHTYV	3.20 ± 0.26	2.53 ± 0.23	1.90 ± 0.16
LZO-5	EGFHL	>4.0	>4.0	>4.0
LZO-6	HAGYSWA	3.53 ± 0.37	2.57 ± 0.24	1.82 ± 0.15
LZO-7	THAWSV	>4.0	>4.0	>4.0
LZO-8	FRHALS	0.19 ± 0.02	0.37 ± 0.04	0.68 ± 0.06
LZO-9	FKEHGY	3.81 ± 0.45	3.02 ± 0.49	3.56 ± 0.34
LZO-10	FSHRGH	2.21 ± 0.23	>4.0	3.16 ± 0.33

a IC_50_ values are shown as mean ± standard error of the mean (SD), from at least three independent experiments.

Nowadays, the mortality of lung cancer is increasing rapidly.^1^ Therefore new therapeutic agents with less toxic effects are currently being explored intensively. It has been reported that natural products are a significant resource for the development of anti-cancer agents. LingZhi is effective against multiple kinds of cancers, including breast, leukemia, and prostate cancers.^7,14^ However, the apoptotic potency of LZOs in lung cancer is not well explored. This is the reason why we chose LZOs and lung cancer for material in the current study. After we got the LZOs, the anti-proliferative potency was evaluated in human lung tumor A549, NCI-H460, and NCI-H23 cell lines.

Interestingly, the MTT results showed that the LZOs inhibited the growth of all the three cancer cells (Table 1). Captivatingly, LZO-3 significantly suppressed the growth of A549 cells in a concentration-dependent manner. Further, strong growth inhibition was found after 48 h, with the IC_50_ value of 0.16 ± 0.02 mM. However, LZO-3 did not affect the growth and viability of human normal pulmonary epithelial cells up to 12 mM at 48 h. Notably, LZO-3 inhibits the growth of A549 cells without affecting cell proliferation in normal human lung cells. So, further study was carried out to explore the anti-proliferative potency of LZO-3 in A549 cells.

### 3.2 LZO-3 induces cell cycle arresting *via* cyclin regulation

To determine whether the anti-cancer potency of LZO-3 has any impact on the cell cycle, the cell cycle distribution was detected. A549 cells were treated as an experimental design, and the DNA content was measured using PI staining, followed by the flow cytometry analysis.

**Fig. 3:**
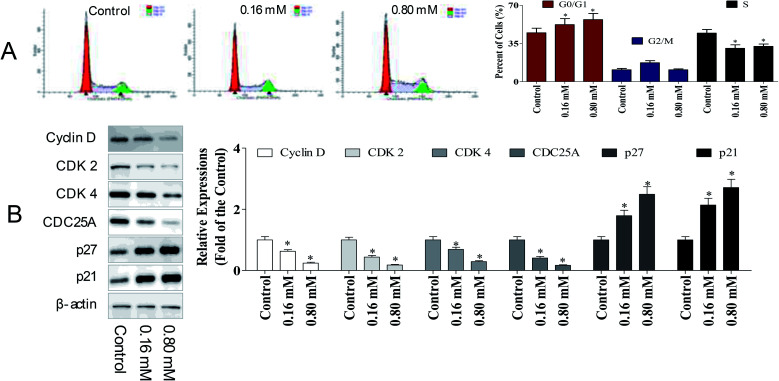
LZO-3 induced cell cycle arrest by modulating cyclins expression. (A) Cell cycle distribution of A549 cells under LZO-3 treatments. LZO-3 increased the cells in G0/G1 population (B) A549 cells were treated with of LZO-3 as an experimental design. western blotting was performed to detect p27, p21, cyclin D, cyclin E, CDK2, and CDK6. β-Actin was used as a loading control. The data are expressed as the mean ± SD of 3 independent experiments. *p < 0.05, **p < 0.01, compared with the control group. Blots were quantified using the ImageJ software.

As shown in Fig. 3A, a dose-dependent increase in the G0/G1 population was observed. Compared to the control group, the G0/G1 population increased by 7.51% and 12.1%, respectively. The accumulation of cells in the G0/G1 population phase is a well-known marker of cell apoptosis. Hence, the cell cycle analysis indicated that the anti-proliferative potency of LZO-3 was associated with cell apoptosis.

To further investigate the molecular basis by which LZO-3 inhibited the G0/G1 transition in tumor cells, we treated cells with LZO-3, and then, analyzed the expression of proteins involved in the cell cycle regulation. We found that the LZO-3 treatment inhibited the cyclin D expression and reduced the expression of CDK2 and CDK4; in contrast, p21 and p27 increased in the HepG2 cells (Fig. 3B). The expression level of the cell division cycle 25A (CDC25A), which acts as an upstream regulator of the CDK/cyclin complex, was significantly inhibited by LZO-3. Notably, the down-regulation of cyclin-D, CDK-2, and increased G0/G1 cell populations confirmed the anti-cancer potency of LZO-3.

### 3.3 LZO-3 triggers apoptosis through an intrinsic caspase pathway

Apoptosis is a sequential genetic program that is involved in a diverse range of cellular processes, including tumor suppression. During the process of carcinogenesis, cancer cells modulate several key proteins and develop resistance against apoptotic death.^15^ Hence, developing anti-cancer agents that promote apoptosis in tumor cells has been intensively investigated. To detect the potency of LZO-3 on apoptosis, A549 cells were treated as an experimental design, and the cell apoptosis was detected by Annexin V/FITC and PI staining.

**Fig. 4:**
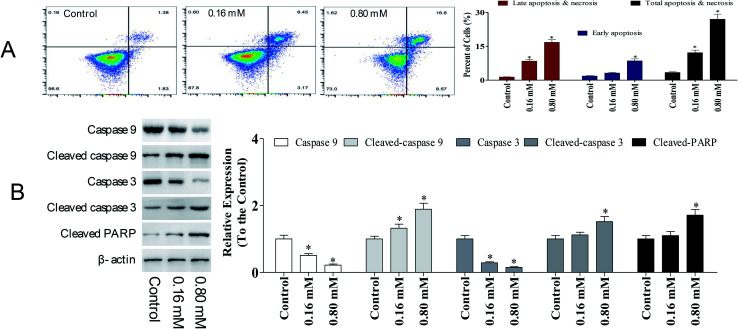
LZO-3 induced caspase-dependent apoptosis in A549 cells. Representative scatter diagrams. (A) Flow cytometric analysis of LZO-3-induced apoptosis in A549 cells using Annexin V-FITC/PI staining. LZO-3 induced cell apoptosis (48 h), which were imaged by using Annexin V/FITC and PI dual staining. (B) LZO-3 induces cells apoptosis by activating the caspases. Equal amounts of whole-cell extracts were separated by 10% SDS-PAGE, electrotransferred onto PVDF membranes, and analyzed by western blotting using the indicated antibodies against proteins related to the caspase-dependent apoptosis. β-Actin was used as a loading control. The data are expressed as the mean ± SD of 3 independent experiments. *p < 0.05, **p < 0.01, compared with the control group.

As shown in Fig. 4A, a significant increase in early apoptotic cells was observed upon LZO-3 treatments. Compared to the control group, the total apoptosis and necrosis rate was increased by 8.80% and 23.6%, respectively. Altogether the results suggest that LZO-3 may promote the cell apoptosis of A549 cells in a dose-dependent manner. Hence, in order to understand the LZO-3 induced apoptosis, the expressions of apoptotic related protein were measured *via* western blotting.

Caspases are the crucial mediators of cell apoptosis. It is well established that both intrinsic and extrinsic apoptotic pathways are executed by different caspases.^16^ Among them, caspase-3 is a frequently activated death protease, catalyzing the specific cleavage of numerous key cellular proteins.^17^ Caspase-9 is involved in the initiation and execution of the intrinsic apoptosis pathway. PARP (poly(ADP-ribose) polymerase) is a family of protein, which gets activated during DNA repair, apoptosis, transcription, and recombination.^18,19^

The expressions of caspase-3, caspase-9, and PARP were evaluated. A significant elevation of cleaved-caspase-3 and 9 was observed during the LZO-3 treatments (Fig. 4B). Moreover, the up-regulation of caspases resulted in the cleavage or inactivation of the PARP protein, which serves as a biochemical marker for the cells undergoing apoptosis.^19^ The cleavage of PARP was prominently observed in A549 cells with LZO-3 treatments. Of note, the results revealed that the anti-cancer action of LZO-3 was associated with the activation of intrinsic apoptosis in A549 cells.

### 3.4 LZO-3 induced apoptosis *via* mitochondrial dysfunction

**Fig. 5:**
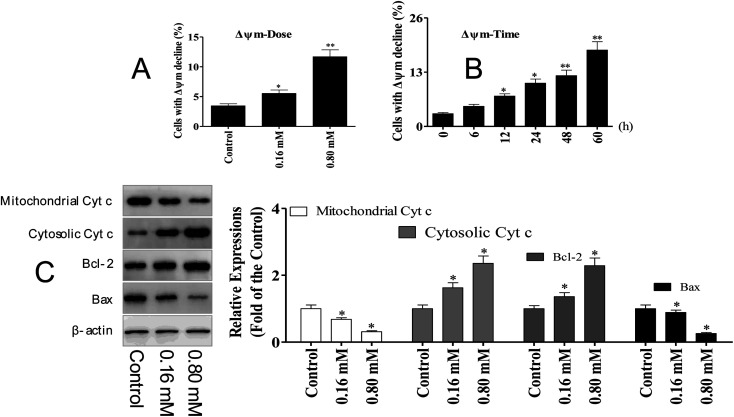
LZO-3 induced apoptosis *via* the mitochondrial pathway. (A and B) Decrease in the mitochondrial potential induced by LZO-3. After treatment with LZO-3, the cells were stained with JC-1 for 15 min and analyzed *via* flow cytometry. (C) A549 cells were treated with different concentrations of LZO-3. Western blotting was performed to detect the cytosolic and mitochondrial levels of the pro-apoptotic proteins cytochrome c, Bcl-2 and Bax. The data are expressed as the mean ± SD of 3 independent experiments. *p < 0.05, **p < 0.01, compared with the control group. Blots were quantified using the ImageJ software.

The activation of caspase-3 and 9 was observed in A549 cells following LZO-3 treatments, which strongly suggested the involvement of the mitochondria-mediated apoptotic pathway. Moreover, the intrinsic apoptosis was activated only upon the loss of the mitochondrial membrane potential (Δ*Ψ*_m_).^20^ Hence, we explored whether the LZO-3 induced apoptosis occurred through the Δ*Ψ*_m_ collapse. A549 cells were treated with LZO-3 as an experimental design and changes in Δ*Ψ*_m_ were measured. The results showed that a time-and dose-dependent Δ*Ψ*_m_ decline was observed in LZO-3 treated cells, and a remarkable disruption was observed at 60 h (Fig. 5A and B). These results clearly indicated that the LZO-3 induced apoptosis was associated with Δ*Ψ*_m_ decline, and the activation of intrinsic apoptosis in A549 cells.

The disruption of mitochondrial membrane potential results in the release of apoptogenic factors in the cytoplasm, which can further execute the caspase cascade pathway.^21^ Mitochondrial dysfunction, as indicated by the dissipation of Δ*Ψ*_m_, could subsequently cause the release of cytochrome c (Cyt c) from mitochondria into the cytosol.^22^ Hence, the cytosolic and mitochondrial Cyt c levels were measured by western blotting. The results showed that LZO-3 treatments resulted in a striking up-regulation of cytosolic Cyt c and down-regulation of mitochondrial Cyt c compared with the control groups (Fig. 5C).

Bcl-2 family of proteins is the key player governing both intrinsic and extrinsic apoptosis through maintaining mitochondrial permeability. It is well established that Bcl-2 family members act as the regulators of mitochondrial membrane permeability, and control the release of apoptotic factors (Cyt c) into the cytosol.^23^ Hence, we detected the expression level of pro-apoptotic and anti-apoptotic proteins upon the LZO-3 treatment by western blotting.

The results showed (Fig. 5C) that LZO-3 treatments significantly suppressed the Bcl-2 expression and strongly increased the Bax expressions. Notably, LZO-3 activates the mitochondria-mediated apoptosis in A549 cells and would be associated with the down-regulation of the Bcl-2/Bax ratio.

### 3.5 Regulation of p53/MDM2 in LZO-3 induced apoptosis

p53 is a tumor suppressor, which plays multiple roles, including the ability to induce cell cycle arrest, DNA repair, senescence, and apoptosis. Hence, the reactivation of the p53 expression is the most promising approach in anti-cancer chemotherapeutics, while MDM2 (murine double minute 2) is the main endogenous negative regulator. This oncoprotein MDM2 binds p53 and negatively regulates the p53 activity *via* the direct inhibition of the p53 transcriptional activity and enhancement of p53 degradation *via* the ubiquitin–proteasome pathway. Inhibiting the p53/MDM2 interactions to restore the p53 activity represents an appealing therapeutic strategy for numerous wild-type p53 tumors with overexpressed MDM2.^10^ Multiple evidence support that excessive ROS accumulation promotes cell apoptosis.^24^ Since, the cell cycle arrest has verified the involvement of p53 during LZO-3 treatment, so we detected the expressions of p53 and MDM2 after the treatments of LZO-3.

**Fig. 6:**
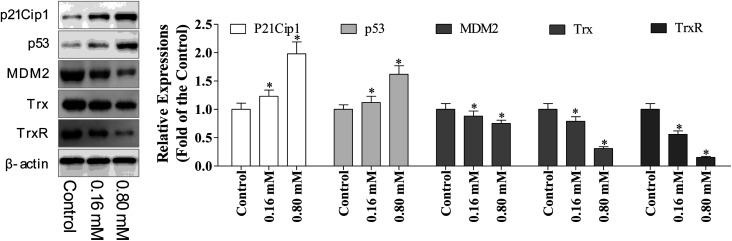
LZO-3 targeted MDM2/p53 state, Trx/TrxR reductase to induce the A549 cells apoptosis. A549 cells were treated with LZO-3 as an experimental design and the protein expression of p53, MDM2, p21Cip1, Trx, and TrxR were measured *via* western blotting. The data are expressed as the mean ± SD of 3 independent experiments. *p < 0.05, **p < 0.01, compared with the control group. Blots were quantified using the ImageJ software.

The results showed (Fig. 6) the strong up-regulation of p53 and down-regulation of MDM2 upon LZO-3 treatments. p21Cip1 comes under the family of CKIs (cyclin-dependent kinase inhibitors), which is activated by p53. Further, a significant elevation of the p53 downstream target protein p21Cip1 was also observed.^25^ Hence, the activation of p53 and p21Cip1 proteins and the down-regulation of MDM2 by LZO-3 substantiated the role of LZO-3 in inducing apoptosis. The down-regulation of the p53 pathway also significantly affected the LZO-3 induced apoptosis in the A549 cells, which was evidenced by the activation of caspases.

### 3.6 LZO-3 disturbs the redox state in A549 cells by targeting Trx/TrxR

The regulation of redox homeostasis is a fundamental phenomenon for normal cellular functions and survival. Growing evidence suggested that the tumor cells have excessive ROS production than normal cells due to their aberrant cell metabolism and continuous cell division.^26^ The hyper-activation of thioredoxin (Trx) and thioredoxin reductase (TrxR) has been reported in numerous cancer cells to regulate their ROS homeostasis, promote cell growth, and induce apoptotic resistance.^27^ From the above experimental results, it is clear that the ROS accumulation plays a crucial role in LZO-3 mediated mitochondrial dysregulation and cell apoptosis. Therefore, we further investigated the role of TrxR and Trx *via* western blotting.

It was interesting to note that decreased expression of both TrxR and Trx (Fig. 6) was found after the LZO-3 treatment, which substantiated the possible deregulation of ROS homeostasis. On the whole, the results strongly supported that LZO-3 modulated the TrxR redox system to induce an excessive ROS accumulation to promote apoptosis. Collectively, the present study disclosed that LZO-3 induced apoptosis *via* the ROS dependent pathway, which was mediated through the Trx/TrxR signaling pathway.

## 4 Conclusion

In the current study, we have demonstrated that LZO-3 possesses anti-cancer potency in A549 cells. Mechanistically, the data showed that LZO-3 induced apoptosis was associated with Δ*Ψ*_m_ collapse, improvement of the MDM2/p53 state, and down-regulation of the Trx/TrxR expressions in A549 cells. To the best of our knowledge, this is the first report demonstrating LingZhi oligopeptide-induced apoptosis accompanied by the redox state disturbance in the tumor cells.

## Conflicts of interest

The authors certify that there is no conflict of interest with any individual/organization for the present work.

## Supplementary Material

RA-010-C9RA10400C-s001
