# Supplementary material for: LingZhi oligopeptides amino acid sequence analysis and anticancer potency evaluation
Source: RSC Adv. 2020 Feb 27;10(14):8377–84. doi: 10.1039/c9ra10400c (PMC9049989; doi:10.1039/c9ra10400c)
Supplement: RA-010-C9RA10400C-s001 [file RA-010-C9RA10400C-s001.pdf]

Electronic Supplementary Material (ESI) for RSC Advances.
This journal is © The Royal Society of Chemistry 2020

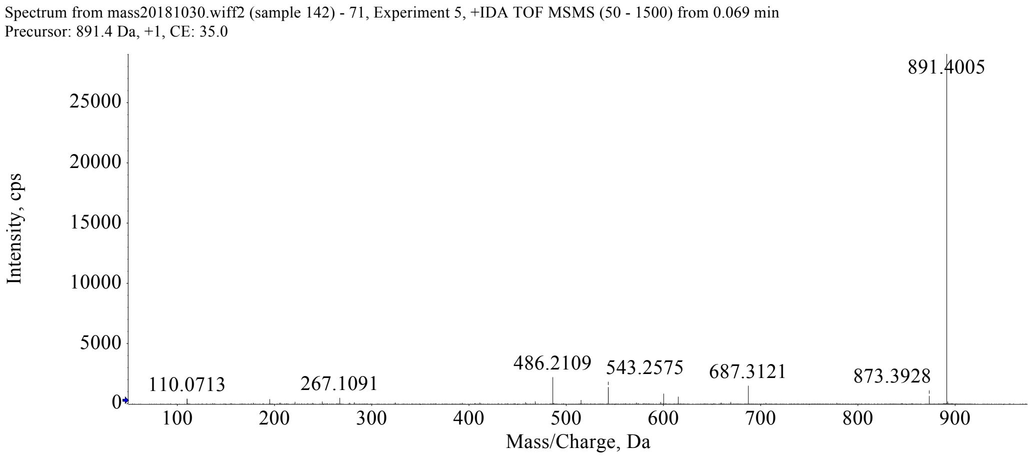

LZO-1

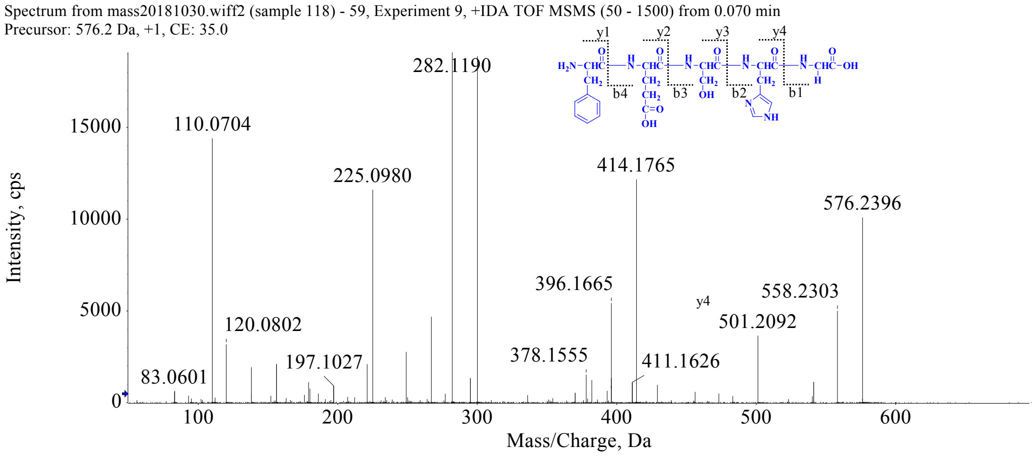

LZO-2

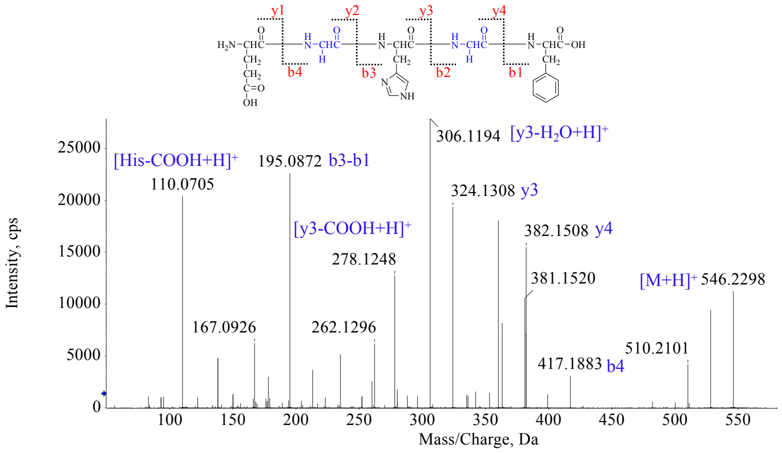

LZO-3

Spectrum from mass20181030.wiff2 (sample 140) - 70, Experiment 5, +IDA TOF MSMS (50 - 1500) from 0.080 min
Precursor: 753.4 Da, +1, CE: 35.0

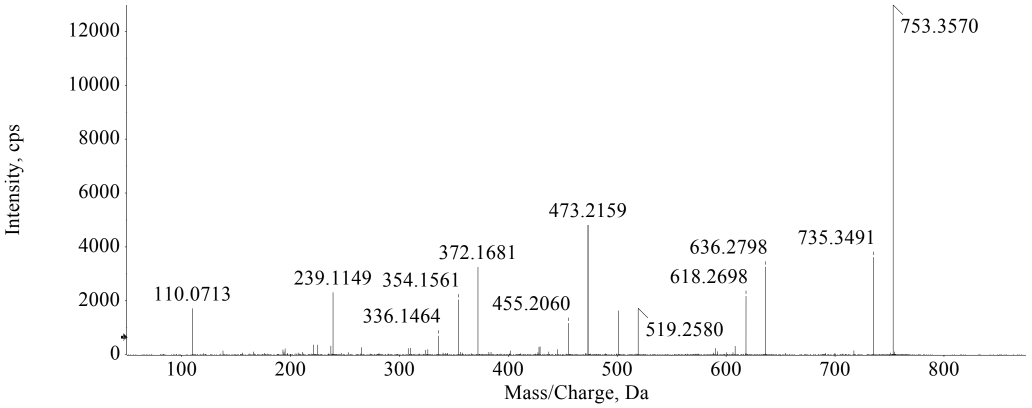

LZO-4

Spectrum from mass20181030.wiff2 (sample 88) - 44, Experiment 8, +IDA TOF MSMS (50 - 1500) from 0.080 min
Precursor: 602.3 Da, +1, CE: 35.0

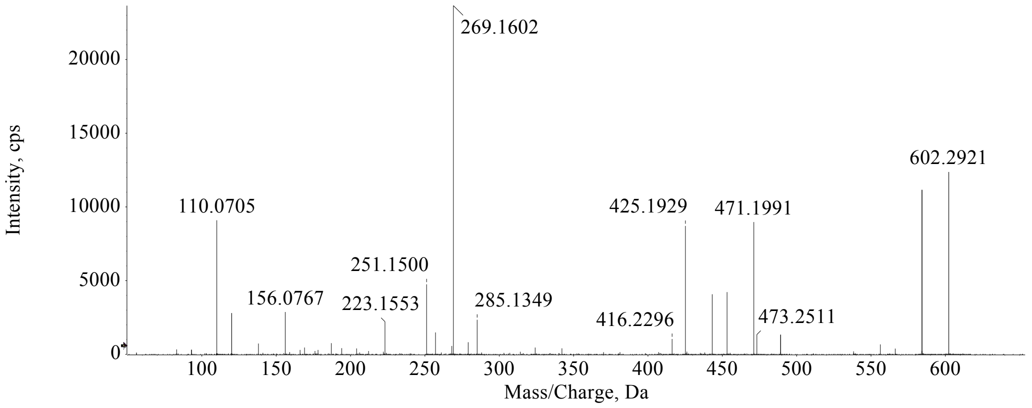

LZO-5

Spectrum from mass20181030.wiff2 (sample 90) - 45, Experiment 9, +IDA TOF MSMS (50 - 1500) from 0.070 min
Precursor: 791.3 Da, +1, CE: 35.0

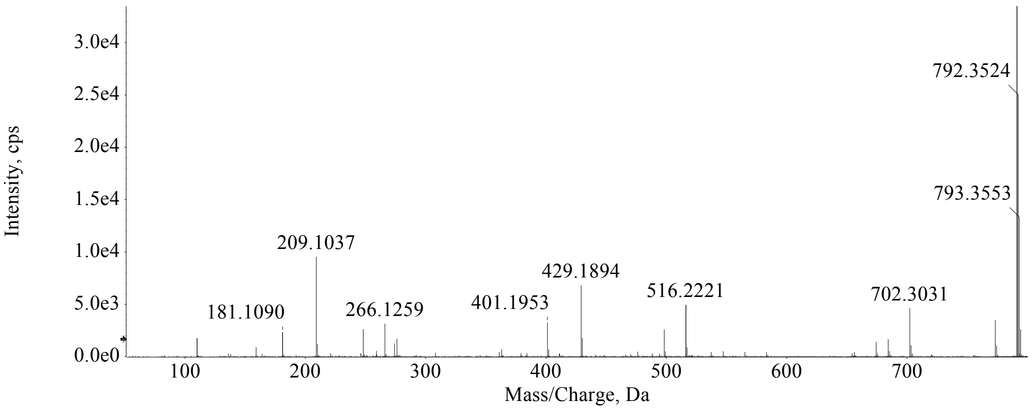

LZO-6

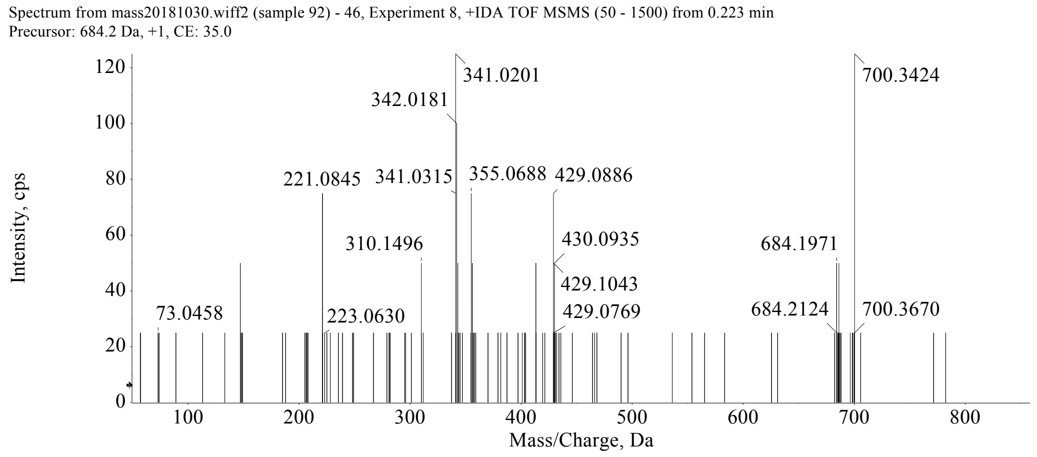

LZO-7

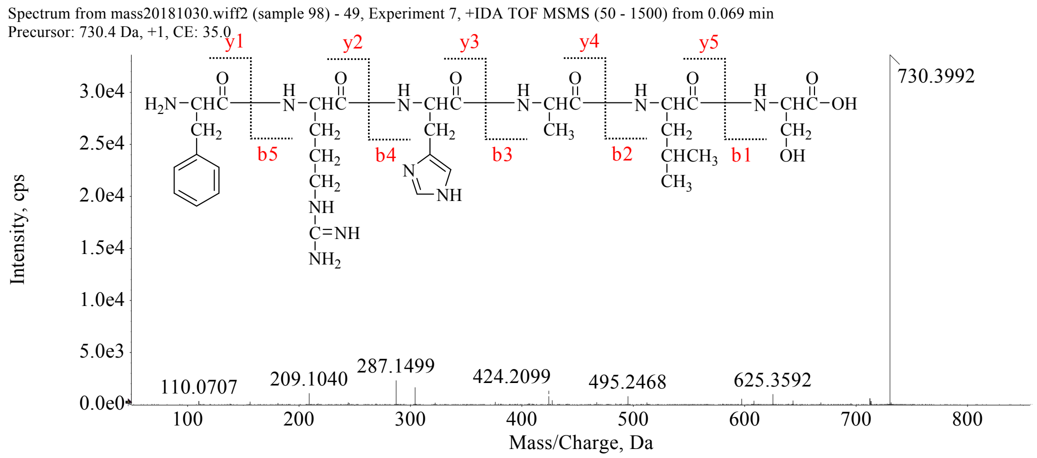

LZO-8

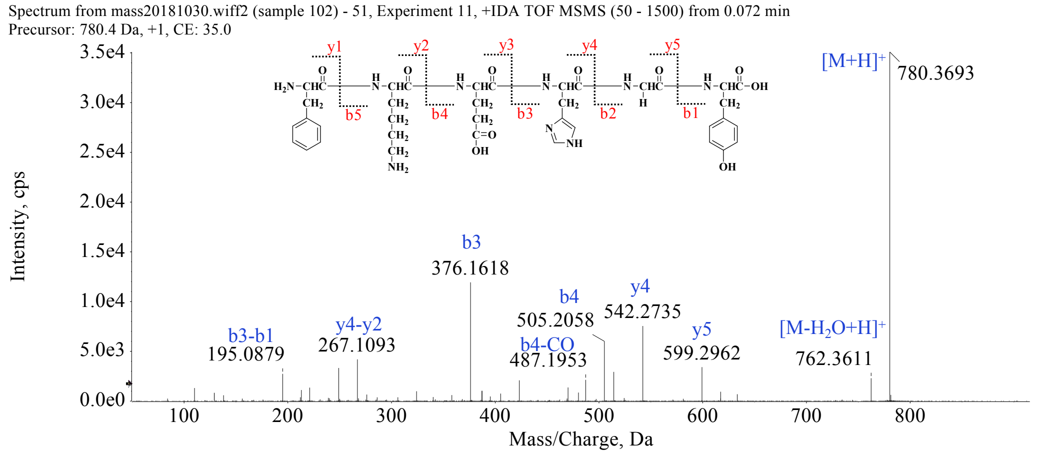

LZO-9

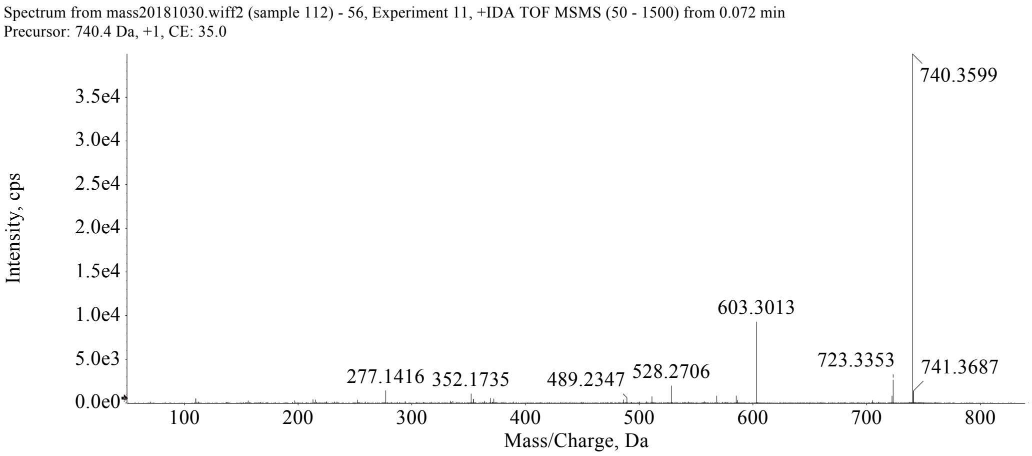

Spectrum from mass20181030.wiff2 (sample 112) - 56, Experiment 11, +IDA TOF MSMS (50 - 1500) from 0.072 min
Precursor: 740.4 Da, +1, CE: 35.0
Intensity, cps
3.5e4
3.0e4
2.5e4
2.0e4
1.5e4
1.0e4
5.0e3
0.0e0
740.3599
603.3013
723.3353
741.3687
277.1416
352.1735
489.2347
528.2706
100
200
300
400
500
600
700
800
Mass/Charge, Da

LZO-10
